# Sex differences of leukocytes DNA methylation adjusted for estimated cellular proportions

**DOI:** 10.1186/s13293-015-0029-7

**Published:** 2015-06-25

**Authors:** Masatoshi Inoshita, Shusuke Numata, Atsushi Tajima, Makoto Kinoshita, Hidehiro Umehara, Hidenaga Yamamori, Ryota Hashimoto, Issei Imoto, Tetsuro Ohmori

**Affiliations:** Department of Psychiatry, Institute of Biomedical Sciences, Tokushima University Graduate School, 3-18-15, Kuramoto, Tokushima, 770-8503 Japan; Department of Human Genetics, Institute of Biomedical Sciences, Tokushima University Graduate School, 3-18-15, Kuramoto, Tokushima, 770-8503 Japan; Department of Bioinformatics and Genomics, Graduate School of Medical Sciences, Kanazawa University, 13-1, Takaramachi, Kanazawa, Ishikawa 920-8640 Japan; Department of Molecular Neuropsychiatry, Osaka University Graduate School of Medicine, Suita, Osaka 5650871 Japan; Department of Psychiatry, Osaka University Graduate School of Medicine, Suita, Osaka 5650871 Japan; Molecular Research Center for Children’s Mental Development, United Graduate School of Child Development, Osaka University, Suita, Osaka 5650871 Japan

**Keywords:** Epigenetics, DNA methylation, Sex, Microarray, Leukocyte, Blood, Cell heterogeneity

## Abstract

**Background:**

DNA methylation, which is most frequently the transference of a methyl group to the 5-carbon position of the cytosine in a CpG dinucleotide, plays an important role in both normal development and diseases. To date, several genome-wide methylome studies have revealed sex-biased DNA methylation, yet no studies have investigated sex differences in DNA methylation by taking into account cellular heterogeneity. The aim of the present study was to investigate sex-biased DNA methylation on the autosomes in human blood by adjusting for estimated cellular proportions because cell-type proportions may vary by sex.

**Methods:**

We performed a genome-wide DNA methylation profiling of the peripheral leukocytes in two sets of samples, a discovery set (49 males and 44 females) and a replication set (14 males and 10 females) using Infinium HumanMethylation450 BeadChips for 485,764 CpG dinucleotides and then examined the effect of sex on DNA methylation with a multiple linear regression analysis after adjusting for age, the estimated 6 cell-type proportions, and the covariates identified in a surrogate variable analysis.

**Results:**

We identified differential DNA methylation between males and females at 292 autosomal CpG site loci in the discovery set (Bonferroni-adjusted *p* < 0.05). Of these 292 CpG sites, significant sex differences were also observed at 98 sites in the replication set (*p* < 0.05).

**Conclusions:**

These findings provided further evidence that DNA methylation may play a role in the differentiation or maintenance of sexual dimorphisms. Our methylome mapping of the effects of sex may be useful to understanding the molecular mechanism involved in both normal development and diseases.

**Electronic supplementary material:**

The online version of this article (doi:10.1186/s13293-015-0029-7) contains supplementary material, which is available to authorized users.

## Background

Sex differences have been widely observed not only in genetics and hormones but also in expression of genes and microRNA [1–4]. DNA methylation, which is most frequently the transference of a methyl group to the 5-carbon position of the cytosine in a CpG dinucleotide, is one of the major mechanisms of epigenetic modifications. This modification plays an important role in gene expression, chromosomal stability, genomic imprinting, X-chromosome inactivation, and mammalian development [5, 6]. Recent genome-wide methylome studies have revealed sex-biased DNA methylation in specific genes on the autosomes of several tissues, such as the blood, brain, and saliva [7–9, 4]. However, researchers have not yet investigated the sex differences in DNA methylation by taking into account cellular heterogeneity, although several studies have demonstrated the effects of cellular heterogeneity on DNA methylation status [10–16], and cell-type proportions may vary by sex.

To reveal sex differences in DNA methylation in human blood, we conducted a genome-wide profiling of DNA methylation by using peripheral leukocytes and then examined sex-biased DNA methylation after correcting the estimated cell-type proportions of each sample.

## Methods

### Subjects

Ninety-three healthy subjects (49 males and 44 females; mean age: 43.6 ± 12.3 years) for our discovery set and 24 healthy subjects (14 males and 10 females, mean age: 35.3 ± 11.9 years) for our replication set were recruited from volunteers who comprised hospital staff, university students, and company employees. There was no significant age difference between male and female groups in both sample sets (*p* > 0.05). All subjects who joined this study were of unrelated Japanese origin and signed written informed consent forms that were approved by the institutional ethics committees of Tokushima University Graduate School and the Osaka University Graduate School of Medicine.

### DNA methylation methods

Genomic DNA was prepared from peripheral blood samples. A bisulfite conversion of 500 ng of genomic DNA was performed with the EZ DNA methylation kit (Zymo Research). DNA methylation levels were assessed with Infinium HumanMethylation450 BeadChips (Illumina Inc.) according to the manufacturer’s instructions. This array’s technical schemes, accuracy, and high reproducibility have been described in previous papers [17–19]. Quantitative measurements of DNA methylation were determined for 485,764 CpG dinucleotides that covered 99 % of the RefSeq genes and were distributed across whole gene regions, including promoters, gene bodies, and 3′-untranslated regions (UTRs). The arrays also covered 96 % of the CpG islands (CGIs) from the UCSC database with additional coverage in CGI shores (0–2 kb from CGI) and CGI shelves (2–4 kb from CGI). DNA methylation data were analyzed using the methylation analysis module within the BeadStudio software (Illumina Inc.). The DNA methylation status of the CpG sites was calculated as the ratio of the signal from a methylated probe relative to the sum of both the methylated and unmethylated probes. This value, known as *β*, ranges from 0 (completely unmethylated) to 1 (fully methylated). For intra-chip normalization of the probe intensities, we performed color balance and background corrections on every set of 12 samples from the same chip by using internal control probes. For quality control, *β* values with detection *p* values ≥0.05 were treated as missing values. Qualified CpG sites used in statistical analyses were defined as follows: 1) autosomal CpGs with no missing values in all subjects; 2) CpGs with no probe single nucleotide polymorphism (SNPs) at minor allele frequencies ≥5 % in the HapMap-JPT population; 3) CpGs with no probe cross-reactivity, and no SNPs at CpG sites and single-base extension sites in a previous paper [20]. The final data set included 345,235 CpG sites (promoter: 152,298; gene body: 104,707; 3′-UTR: 10,306; intergenic region: 77,924; CpG island: 117,528; CpG island shore; 84,341; CpG island shelf: 30,207; others: 113,159). We deposited genome-wide DNA methylation data to the Gene Expression Omnibus (GEO) of the National Center for Biotechnology Information under the accession number GSE67393.

### Statistical analysis

The cell-type proportions (CD4 + T cell, CD8 + T cell, CD56 + NK cell, CD19 + B cell, CD14 + monocyte, and granulocyte) for each of the samples were estimated using a published algorithm [21, 22] implemented in an R-package “Minfi,” as we had done in our previous study [15]. Surrogate variable analysis (SVA), which is a method for modeling the potential confounding factors that may or may not be known, including technical factors such as batch effects, can increase the biological accuracy and reproducibility of analyses in microarray studies [23, 24]. We used SVA to identify the potential confounding factors in our microarray data as surrogate variables (SVs). Then, we examined the influences of sex on DNA methylation with a multiple linear regression analysis after adjusting for age, significant SVs (8 SVs in the first set and 6 SVs in the replication set), and the estimated 6 cell-type proportions, as in a previous study [8]. Bonferroni correction was applied at the 0.05 level for multiple testing (nominal *p* value of 1.44 × 10^−7^). The gene-ontology analysis was performed with the Database for Annotation, Visualization and Integrated Discovery (DAVID) [25].

## Results

### Estimated cell-type proportions between males and females

**Fig. 1:**
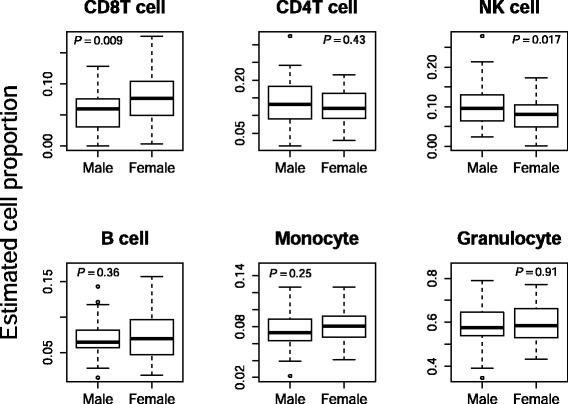
Average estimated cellular proportions of male and female groups. The *y axis* is each of average estimated cellular proportions of CD8 + T cell, CD4 + T cell, CD56 + NK cell, CD19 + B cell, CD14 + monocyte, and granulocyte. Significant differences between the two groups were observed in 2 cell types (CD8 + T cell and CD56 + NK cell) (Welch’s *t* test *p* < 0.05)

We estimated 6 cell-type proportions using “Minfi”, a flexible and comprehensive bioconductor package for the analysis of Infinium DNA methylation microarrays developed by Aryee et al. [21]. The average estimated cellular proportions of the male and female groups are shown in Fig. 1. Of the 6 cell types, 2 (CD8 + T cell and CD56 + NK cell) showed small but significant differences between the two groups (Welch’s *t* test *p* < 0.05), which could be confounding factors in determining sex-differential DNA methylation sites.

### Sex differences in DNA methylation in the blood

**Fig. 2:**
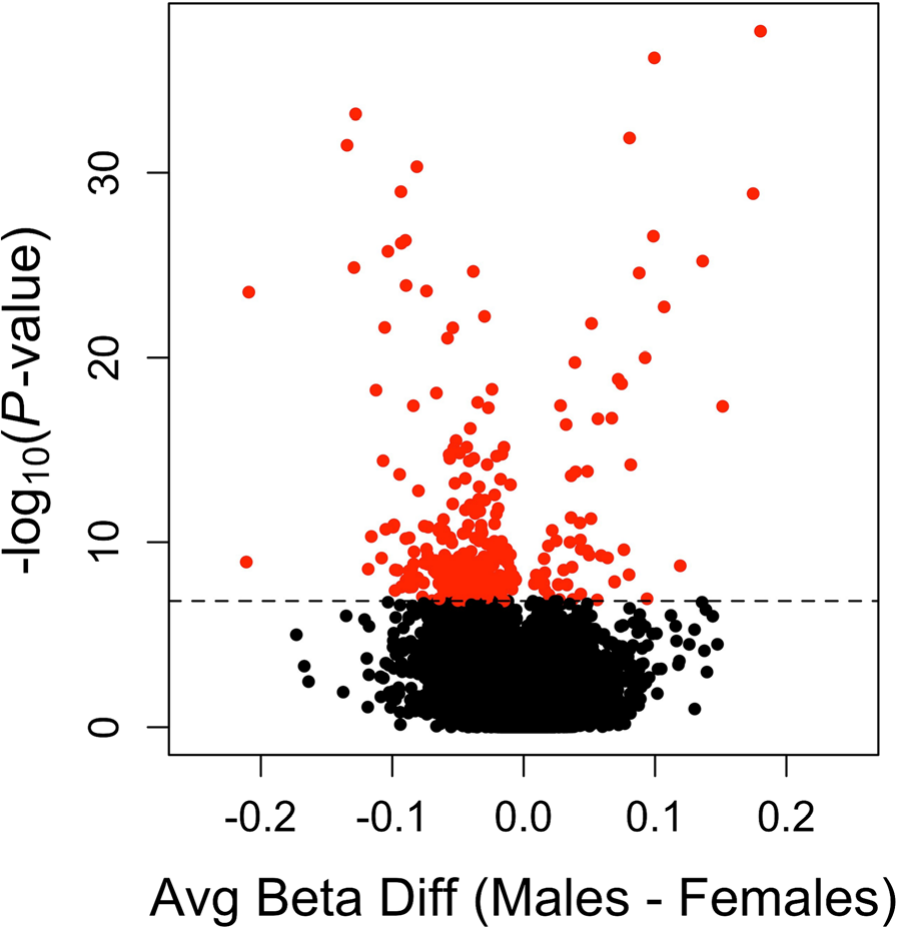
Volcano plots of differentially methylated CpG sites between males and females. This volcano plot shows the result of genome-wide DNA methylation differences between 49 males and 44 females after adjusting for the estimated cell mixture proportions. Average beta difference (males-females) is shown on the *x axis*. Log_10_-converted *p* value is shown on the *y-axis*. CpG loci that showed a *p* value of less than 5 % after Bonferroni correction are colored *red*. Significant sex differences in DNA methylation were observed at 292 CpG sites (*p* < 1.44 × 10^−7^)

**Fig. 3:**
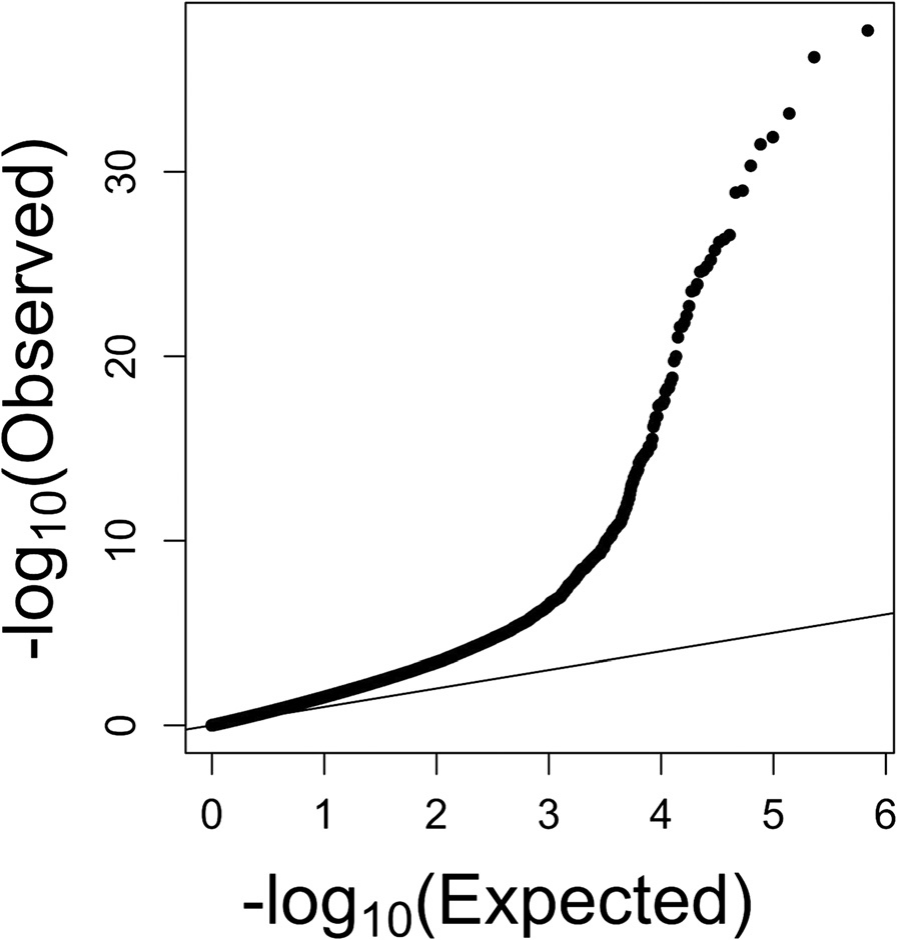
Quantile-quantile (Q-Q) plot of DNA methylation between males and females. The *x axis* is the expected −log_10_P value, and the *y axis* is the observed −log_10_P value. This Q-Q plot shows a deviation of the observed from the expected, providing evidence of DNA methylation differences between males and females at numerous CpG sites

The DNA methylation levels of 93 subjects were evaluated using Infinium HumanMethylation450 BeadChips, and then the sex differences of these levels between 49 males and 44 females were assessed using a multiple linear regression analysis after adjusting for age, the estimated cell-type proportions, and the SVs identified in our SVA. Of the 345,235 CpG sites, significant sex differences in DNA methylation were observed at 292 CpG sites (nominal *p* < 1.44 × 10^−7^, Additional file 1). When we analyzed array data without adjusting for the estimated cell-type proportions, significant sex differences were observed at 417 CpG sites (Additional file 2), and 270 sites were common between the results from the adjusted and un-adjusted analyses. The reduction in differentially methylated sites after cell proportion adjustment suggests that the present statistical analysis has the ability to lower false-positive detections of sex-differential DNA methylation sites. Figure 2 shows volcano plots of differentially methylated CpG sites between males and females. Figure 3 shows a quantile-quantile (Q-Q) plot of −log_10_P values, which deviates from their expected values under the null hypothesis. Of the 292 CpG sites, 237 sites (81.2 %) showed higher methylation in females than in males. Table 1 lists the top 20 CpG sites that showed significant sex differences. When these 292 CpG sites were classified into 4 different categories according to their locations in the genes (promoter, gene body, 3′-UTR, and intergenic region), 139 sites (47.6 %) were located in the promoter regions, 59 sites (20.2 %) in gene bodies, and 2 sites (0.7 %) in 3′-UTRs. When these 292 CpG sites were classified into 4 categories according to the CpG content in the genes (CGI, CGI shore, CGI shelf, and others), 139 sites (47.6 %) were located in the CGIs, 10 sites (3.4 %) in CGI shores, and 76 sites (26 %) in CGI shelves.

**Table 1:** Top 20 autosomal CpG sites with significant sex differences

						First set				Second set			
Target ID	UCSC RefGene name	Chromosome	Position^a^	Relation to UCSC CpG island	UCSC RefGene group	Mean *β* value of male	Mean *β* value of female	Sex average difference of *β* value	Sex *p* value	Mean *β* value of male	Mean *β* value of female	Sex average difference of *β* value	Sex *p* value
cg12691488		1	2.4E +08	CGI	Intergenic	0.352	0.171	0.180	2.26E-38	0.263	0.111	0.152	2.11E-03
cg03618918		1	1.6E +08	Others	Intergenic	0.790	0.691	0.099	6.38E-37	0.825	0.745	0.079	3.04E-03
cg17238319	RFTN1	3	1.6E +07	Others	Gene body	0.660	0.788	−0.128	6.88E-34	0.703	0.816	−0.113	5.19E-02
cg25304146	WBP11P1	18	3E +07	Others	Gene body	0.630	0.550	0.081	1.31E-32	0.704	0.604	0.100	5.78E-02
cg03691818	KRT77	12	5.3E +07	Others	Gene body	0.043	0.178	−0.134	3.25E-32	0.052	0.254	−0.202	7.13E-04
cg17232883		11	5.9E +07	Others	Intergenic	0.076	0.157	−0.081	4.72E-31	0.056	0.123	−0.067	1.42E-04
cg25568337	ARID1B	6	1.6E +08	Others	Promoter	0.164	0.258	−0.093	1.07E-29	0.152	0.297	−0.145	1.15E-04
cg04946709	LOC644649	16	6E +07	CGI	Gene body	0.805	0.631	0.175	1.37E-29	0.823	0.499	0.324	8.79E-05
cg22266749	COL25A1	4	1.1E +08	CGI	Promoter	0.192	0.093	0.099	2.77E-27	0.229	0.107	0.122	1.52E-02
cg12177922	HAX1	1	1.5E +08	CGI	Promoter	0.207	0.297	−0.090	4.68E-27	0.161	0.236	−0.074	3.68E-02
cg23719534		15	1E +08	CGI	Intergenic	0.859	0.952	−0.093	6.67E-27	0.858	0.949	−0.091	3.87E-03
cg20299935		17	2.2E +07	Others	Intergenic	0.708	0.811	−0.103	1.84E-26	0.740	0.813	−0.073	8.56E-01
cg12052203	B3GNT1	11	6.6E +07	CGI	Promoter	0.172	0.035	0.136	6.19E-26	0.210	0.050	0.160	1.23E-03
cg06710937		13	2.3E +07	CGI	Intergenic	0.062	0.191	−0.129	1.39E-25	0.036	0.173	−0.137	5.72E-04
cg23814743	NICN1	3	4.9E +07	CGI	Promoter	0.239	0.277	−0.038	2.25E-25	0.121	0.158	−0.038	1.81E-03
cg15817705		1	2.1E +08	CGI shore	Intergenic	0.750	0.662	0.088	2.73E-25	0.759	0.683	0.076	3.01E-02
cg03218192	AP2B1	17	3.4E +07	Others	Promoter	0.276	0.365	−0.089	1.30E-24	0.228	0.344	−0.116	2.43E-03
cg07852945	TLE1	9	8.4E +07	CGI	Promoter	0.103	0.177	−0.074	2.58E-24	0.047	0.137	−0.090	1.83E-04
cg23256579	PRR4	12	1.1E +07	Others	Promoter	0.422	0.631	−0.209	2.97E-24	0.363	0.573	−0.210	4.33E-03
cg25294185	RNASEH2C	11	6.5E +07	CGI	Gene body	0.171	0.064	0.107	1.88E-23	0.162	0.086	0.076	6.48E-01

^a^Positions refer to Genome Research Consortium human genome build 37 (GRCh37)/UCSC human genome 19 (hg19)

### Gene-ontology analysis

We used DAVID to perform a gene-ontology analysis of the genes, which showed significant sex differences in DNA methylation, and revealed enrichment of genes related to secretion and secretion by cell. Table 2 lists the significant gene-ontology categories.

**Table 2:** Gene-ontology analysis of the genes which showed significant sex differences in DNA methylation in this study (*p* < 0.01)

Category	Term	Gene count (%)	*p* value	Fold enrichment
GOTERM_CC_FAT	GO:0031965~ nuclear membrane	5 (3.36)	2.49.E-03	8.70
GOTERM_CC_FAT	GO:0031301~ integral to organelle membrane	6 (4.03)	2.74.E-03	6.17
GOTERM_CC_FAT	GO:0012505~ endomembrane system	15 (10.07)	2.84E-03	2.43
GOTERM_CC_FAT	GO:0005635~ nuclear envelope	7 (4.70)	5.42.E-03	4.31
GOTERM_BP_FAT	GO:0032940~ secretion by cell	7 (4.70)	5.85.E-03	4.25
GOTERM_CC_FAT	GO:0031300~ intrinsic to organelle membrane	6 (4.03)	5.96.E-03	5.14
GOTERM_BP_FAT	GO:0046903~ secretion	8 (5.37)	9.30.E-03	3.36

### Validation of sex differences in an independent set of samples

DNA methylation levels were measured in an independent cohort of 14 males and 10 females using the same Illumina DNA methylation arrays. Of the top 20 differentially methylated CpG sites between males and females in the first set, the same directions (male > female or male < female) were observed at all CpG sites, and significant sex differences were also observed at 16 sites in the replication set (*p* < 0.05) (Table 1). Of the 292 differentially methylated CpG sites in the first set, significant sex differences were also observed at 98 sites in the replication set (*p* < 0.05).

## Discussion

In this study, we conducted a genome-wide DNA methylation profiling of the peripheral leukocytes from non-psychiatric subjects using Infinium HumanMethylation450 BeadChips and identified sex-biased genes on autosomes by adjusting for the estimated cell-type proportions. This blood study is the first to reveal sex differences in DNA methylation by taking into account cellular heterogeneity of blood in the analysis.

We revealed that most of significant loci (81.2 %) showed higher DNA methylation in females than in males. This finding is consistent with the results of previous studies [4, 7–9]. However, the explanation for this phenomenon is unclear. Gene-ontology analysis of biological process revealed that genes with sex differences in DNA methylation on autosomes were related to secretion and secretion by cell. Of these 8 secretion-related genes, 5 genes (*FKBP1B*, *SCIN*, *SMPD3*, *STEAP2*, and *TRIM36*) has been associated with prostate cancer and hyperplasia [26–30]. These results may suggest some hormone-related genes are sex-differentially regulated, perhaps via methylation.

To date, two genome-wide methylome studies have examined sex-biased DNA methylation using Illumina Infinium HumanMethylation450 BeadChips [4, 9]. When we compared with the 614 sex-biased differential CpG sites on autosomes identified in a previous study using the human prefrontal cortex tissues [4], these CpG sites identified by Xu et al. were significantly enriched for those sites identified in the present study (common CpG site: 93 vs. 293, un-common CpG site: 521 vs. 344,942, odds ratio (OR) = 210; 95 % confidence intervals (CIs), 163–269; Fisher exact test *p* < 0.05). When we compared with the top 20 sex-biased differential CpG sites on autosomes in the study of Xu et al. [4], we observed common sex-biased DNA methylation at 17 CpG sites which covered 14 distinctive genes (*ARID1B*, *C6orf108*, *GLUD1*, *H3F3A*, *KRT77*, *SCIN*, *TFDP1*, *WBP11P1*, *YARS2*, and *ZNF69*) in our blood study. These results suggest that sex-biased DNA methylation on autosomes in the brain is also observed in peripheral blood in specific genes, although tissue-specific differences in DNA methylation have been reported [31, 32]. *ARID1B*, which is a member of the SWI/SNF-A chromatin remodeling complex, has been implicated in intellectual disability and autism spectrum disorders [33, 34]. *GLUD1*, which plays a role at glutamatergic synapses [35], has been implicated in schizophrenia [36]. *H3F3A*, which encodes the replication-independent histone 3 variant H3.3, has been implicated in glioblastoma [37, 38].

When we compared with the 564 sex-biased differential genes on autosomes identified in a previous study using the human blood mononuclear cells from a high-aged cohort (over 95 years old) [9], we observed common sex-biased DNA methylation in only 15 genes (*AGAP11*, *ANKRD11*, *C15orf29*, *HOXC4, HOXC5*, *HOXC6*, *MACROD1*, *NOTCH4*, *NSD1*, *OSTalpha*, *PEX10*, *PTPRN2*, *SHANK3*, *TFDP1*, and *UNC84A*) in our study. This difference between studies might be due to the large difference in subjects’ mean age and the fact that Sun et al. did not correct for sex-differential cell-type proportions. Both age and cell-type proportion are well known to be major confounding factors in DNA methylation [12, 16]. However, sex-biased genes identified by Sun et al. were significantly enriched for those genes identified in the present study (common gene: 15 vs. 193, un-common gene: 549 vs. 19,533, OR = 2.8; 95 % CI, 1.5–4.7; Fisher exact test *p* < 0.05). Mai and colleagues (2010) has demonstrated *HoxC4*-mediated regulation of activation-induced cytosine deaminase expression, as enhanced by estrogen, and has suggested a possible role of this homeodomain transcription factor in mediating immunopotentiation in gestation and neonatal and adult life [39].

There are several limitations to the present study. First, our sample size was not large. Replication studies with larger samples will be needed. Second, the cellular proportions were created by a bioinformatics tool, so these were not based on direct observation of the relative numbers of cells in the sample. Furthermore, experimental noises may be increased due to the circular use of DNA methylation data, as these data are used first to define cell-type proportions, which are then used as covariates in the differential methylation analysis. Cell-type-specific studies will be needed. Third, we did not take other confounding factors, such as smoking or body mass index, into consideration in our analysis, which may affect DNA methylation status [40, 41], because these information were not collected in the present study.

## Conclusions

In summary, we identified sex-biased DNA methylation at numerous CpG sites on autosomes by conducting a comprehensive DNA methylation profiling of blood and by adjusting for estimated cellular proportions. These findings provided further evidence that DNA methylation may play a role in the differentiation or maintenance of sexual dimorphisms, and our methylome mapping of the effects of sex may be useful to understanding the molecular mechanism involved in normal development and diseases.

## Additional files

Additional file 1:
**292 CpG sites which showed significant gender differences in DNA methylation at 5 % Bonferroni correction.**
Additional file 2:
**417 CpG sites which showed significant gender differences in DNA methylation at 5 % Bonferroni correction.**

## Abbreviations

CGIs: CpG islands
DAVID: Database for Annotation, Visualization and Integrated Discovery
SNPs: single nucleotide polymorphism
SVA: surrogate variable analysis
SVs: surrogate variables
UTRs: 3′-untranslated regions
